# Comparing imaging capabilities of spectral domain and swept source optical coherence tomography angiography in healthy subjects and central serous retinopathy

**DOI:** 10.1186/s40662-018-0113-2

**Published:** 2018-08-08

**Authors:** Fupeng Wang, Qinqin Zhang, Anthony J. Deegan, Jun Chang, Ruikang K. Wang

**Affiliations:** 1 Department of Bioengineering, University of Washington, Seattle, Washington 98105 USA; 2 School of Information Science and Engineering, Shandong University, Jinan, 250100 China

**Keywords:** Optical coherence tomography, Spectral domain OCTA, Swept source OCTA, Central serous retinopathy, Signal strength, Central serous volume

## Abstract

**Background:**

There are two forms of system implementation of optical coherence tomography angiography (OCTA) in ophthalmic imaging, i.e., spectral domain (SD-) and swept source OCTA (SS-OCTA). The purpose of this paper is to compare the SD-OCTA and SS-OCTA for elucidating structural and vascular features associated with central serous retinopathy (CSR), and to evaluate the effects of CSR on SD- and SS-OCTA’s imaging capabilities.

**Methods:**

Normal subjects and CSR patients were imaged by SD- and SS-OCTA using 3 × 3 mm and 6 × 6 mm scan patterns. OCT signal strengths at the superficial retina, deep retina, Sattler’s layer and Haller’s layer were used to compare the ability of SD- and SS-OCTA to image structural features. In addition, the ability to acquire angiograms were discussed by evaluating retinal vessel density. Central serous volume (CSV) was measured and it was correlated with difference in signal strengths (∆S) between two OCTA devices.

**Results:**

Seven normal eyes and seven diseased eyes were recruited. Results showed no significant differences between SD- and SS-OCT in detecting structural features of the retinal layer according to the paired t-test. However, when imaging the Sattler’s layer for normal eyes, a significant difference is found between SD- and SS-OCT (*p* < 0.0001 for 3 × 3 mm scan, and *p* = 0.0002 for 6 × 6 mm); while for CSR eyes, the corresponding values were *p* < 0.0001 and *p* = 0.0003, respectively. At Haller’s layer for normal eyes, the corresponding values were *p* = 0.0004 and *p* = 0.0014; and for CSR eyes, *p* = 0.0004 and *p* < 0.0001, respectively. A strong correlation between *∆S* and CSV was observed in the Sattler’s layer (3 × 3 mm – *p* = 0.0031 and *R*^*2*^ = 0.951; 6 × 6 mm – *p* = 0.0075 and *R*^*2*^ = 0.911) and Haller’s layer (3 × 3 mm – *p* = 0.0026 and *R*^*2*^ = 0.955; 6 × 6 mm – *p* = 0.0013 and *R*^*2*^ = 0.972).

**Conclusions:**

The results suggest no differences between SD- and SS-OCTA for imaging the retinal layers however, when imaging beyond retinal layers, SS-OCTA appears advantageous in detecting returning signals. In CSR cases, the CSV may have an impact on sub-CSR tissue imaging and appears to have more impact on SD- than SS-OCTA.

## Background

Central serous retinopathy (CSR), characterized by serous retinal detachment in the macular area, is an eye disease commonly found in young to middle-aged adults [1, 2]. Typically, the disease first presents itself through blurred or distorted vision caused by the accumulation of fluid resulting from retinal detachment. Currently, CSR diagnosis is commonly initiated through bio-microscopy and confirmed by fluorescein angiography (FA) however, the requirements for intravenously injected dye and the associated side-effects of such a dye have prompted the search for an alternative diagnostic technique. Additionally, while CSR can spontaneously resolve within several months without the need for treatment [3], a number of complications are known to occur alongside the disease such as bullous retinal detachment, retinal pigment epithelial atrophy and choroidal neovascularization (CNV) [4–6]. The ability to diagnose such cases becomes increasingly difficult in the presence of CSR because achieving good quality images is difficult for many conventional imaging modalities. Therefore, the need exists for a non-invasive imaging tool that can visualize both CSR and complications.

As a non-invasive, depth-resolved imaging technology [7, 8], optical coherence tomography (OCT) has already been successfully employed to elucidate the pathological features of CSR [9–12]. In addition to structural details, blood flow imaging is also interesting and necessary in some studies of CSR [13, 14], which can be performed using OCT-based angiography (OCTA), a new imaging extension of OCT that provides blood flow information via motion contrast created by moving red blood cells within active vessels [15–17]. While this technique has the potential to significantly improve our diagnostic capacity for CSR cases and others, it still has room for improvement. To further refine OCTA and its potential for clinical uses, various configurations and reiterations have been developed. A number of studies have been conducted to compare two of the most commonly used system designs, spectral domain (SD)- and swept source (SS)-OCTA, using various pathological features such as CNV [18, 19] and choroidal thickening [20–22]. Thus far, all have shown SS-OCTA to be the superior technology when imaging deep choroid due to its longer wavelength and better sensitivity roll-off [21, 23]. There are, however, no prior studies that we are aware of that has used CSR as a model for comparative purposes, nor any studies that have used signal strength as a comparative tool. CSR offers a particularly interesting case for comparing OCTA systems because central serous volume (CSV), a common feature of the disease, extends the optical path for penetrating light, making it more difficult to detect the returning signal from deep tissues. This characteristic makes it necessary to evaluate how CSR would affect both SD- and SS-OCTA when imaging deep retinal layers.

In this study, we conducted a comparison between SD- and SS-OCTA’s capability of providing structural and angiographic information. The metrics of OCT signal strength and vessel density obtained from CSR and normal subjects were used in the comparison. For CSR cases, CSV was calculated as a parameter to evaluate how the CSV, respectively, affects SD- and SS-OCTA’s capability when imaging beyond the retinal layers.

## Methods

### Patient enrollment and scanning protocol

Normal volunteers and CSR patients were recruited and imaged by both the SD- and SS-OCTA systems. The diagnosis of CSR was defined as an idiopathic detachment of the neurosensory retina on slit-lamp observation secondary to one or more focal leaks at the level of the retinal pigment epithelium (RPE) layer on FA. An initial scan size of 3 × 3 mm was used to provide optimal scanning parameters however, given the varying sizes of CSR lesions, 3 × 3 mm scans could not cover the whole lesion therefore, an additional scan pattern of 6 × 6 mm was used. For continuity, both devices were used at the same visit for each subject. This study was conducted under an investigational clinical study approved by the Institutional Review Board of the University of Washington, and informed consent was obtained from each subject prior to imaging. The study followed the tenets of the Declaration of Helsinki and was in compliance with the Health Insurance Portability and Accountability Act.

SD-OCTA was performed using a commercially available AngioPlex instrument (Carl Zeiss Meditec Inc., Dublin, USA) that employed a light source with a central wavelength of 840 nm and a bandwidth of 45 nm. The imaging speed was 68 kHz A-line rate, axial resolution was ~ 5 μm in tissue and lateral resolution was ~ 15 μm. Two scanning patterns were used to collect the OCT structure and angiogram: 3 × 3 mm and 6 × 6 mm. For the 3 × 3 mm scans, 245 A-lines produced one B-scan, and B-scans were acquired at 245 different spatial locations and repeated four times at each location. This scanning protocol resulted in a uniform spacing of 12.2 μm between pixels in the final *en face* projected OCTA images. For the 6 × 6 mm scans, 350 A-lines produced one B-scan, and B-scans were acquired at 350 different spatial locations and repeated two times at each location, resulting in a uniform spacing of 17.1 μm between pixels in the final *en face* projected OCTA images.

SS-OCTA data were acquired using a PlexElite 9000 device (Carl Zeiss Meditec Inc., Dublin, USA), which had a central wavelength of ~ 1050 nm, a bandwidth of 100 nm, and a 100 kHz A-line rate. Axial resolution was ~ 5 μm in tissue while lateral resolution was ~ 14 μm. Again, two scanning patterns were used to image subjects. For the 3 × 3 mm scan, a 300 × 300 pixel-array was used (10 μm spacing between pixels), while the 6 × 6 mm scan used a 500 × 500 pixel-array (12 μm). The repetition strategy was the same as that used for SD-OCTA: four repetitions in 3 mm × 3 mm scans and two repetitions in 6 × 6 mm scans. Both SD-OCTA and SS-OCTA datasets were processed using the complex optical microangiography (OMAG) algorithm [24–27] to generate angiograms.

### Normalization and segmentation

Since the OCT reflectance signal is dependent on how much energy is impinging on the tissue surface, it requires normalizing OCT scans acquired from both systems to make a fair comparison. In this study, the SD- and SS-OCTA data from a single subject was normalized, in which we assumed that the retinal tissue was irradiated with the same total light energy of unity for both the SD- and SS-OCT. Assuming the light absorption is negligible we then may approximate the total light energy by summing all the backscattered light (i.e. OCT signals) from the scanned tissue volume, *S*_*sum*_. Thus, the normalized OCT signal, *S*_*norm*_*(x,y,z)*, can be expressed as:

1 $$ {S}_{norm}\left(x,y,z\right)=\frac{S_m\left(x,y,z\right)}{S_{sum}} $$

where *S*_*m*_*(x,y,z)* is the measured voxel signal located at (x,y,z) position within the 3D OCT scan*.*

**Fig. 1:**
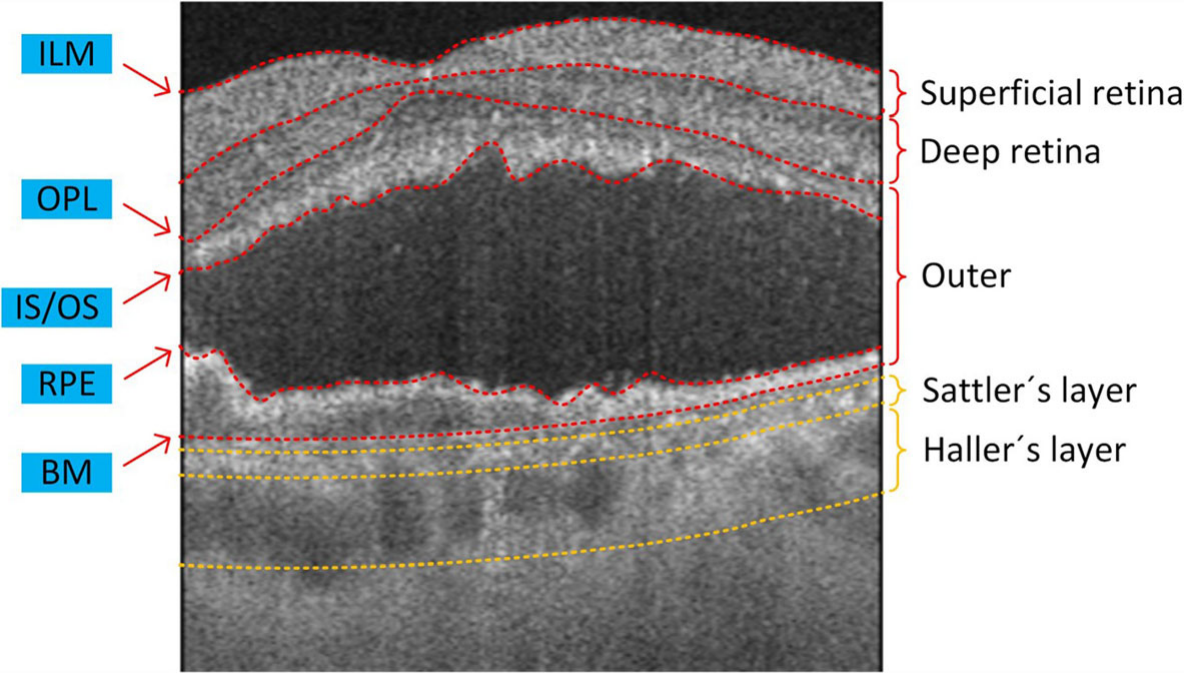
Segmentation layers used throughout this study. ILM: inner limiting membrane, OPL: outer plexiform layer, IS/OS: photoreceptor inner segment and outer segment layers, RPE: retinal pigment epithelium layer, BM: Bruch’s membrane

To evaluate and compare the ability of the system to detect signal at different depths, depth resolved layers in the retina and choroid were segmented by a semi-automatic segmentation software [28] (Fig. 1). Here, the retinal layer is defined as the region from the inner limiting membrane (ILM) to the outer plexiform layer (OPL). Superficial retinal layer is defined as the region from the ILM to the inner plexiform layer (IPL) inclusive. Deep retinal layer is defined as the region from the inner nuclear layer (INL) to the OPL inclusive, and the outer retinal space is defined as the region from the OPL to Bruch’s membrane (BM). Sattler’s and Haller’s layers are defined as medium- and larger-sized vessel layers, respectively [29, 30]. However, automatic segmentation of Sattler’s and Haller’s layers may lead to failure of the comparison in our study because there is no evidence that automatic segmentation would give the same results between SD- and SS-OCTA. Instead, fixed thickness was used to manually segment Sattler’s and Haller’s layers considering the published thickness results [29, 30] (Fig. 1). The CSV is defined as the region from the photoreceptor inner segment and outer segment layers (IS/OS) to the RPE.

### Signal strength calculations

Signal strength of structural images in different depths were calculated to compare the abilities of structural imaging between SD- and SS-OCTA. For each of the eyes scanned, both the 3 × 3 mm and 6 × 6 mm scans from the SD-OCTA and SS-OCTA were analyzed. Four layers (superficial and deep retinal layers, Sattler’s and Haller’s layers) were segmented, as mentioned above. The signal strength of each layer was defined using Eq. (2):

2 $$ {\boldsymbol{S}}_{\boldsymbol{sd}},{\boldsymbol{S}}_{\boldsymbol{ss}}=\frac{{\boldsymbol{S}}_{\boldsymbol{layer}\_\boldsymbol{sum}}}{{\boldsymbol{N}}_{\boldsymbol{layer}\_\boldsymbol{voxel}}} $$

where ***S***_***sd***_ and ***S***_***ss***_ represent the signal strength of each layer from SD- and SS-OCT, respectively. ***S***_***layer***_***sum***_ is the summation of all backscattered signal within each layer and ***N***_***layer***_***voxel***_ represents the number of voxels in that layer.

### Retinal layer vessel density measurements

**Fig. 2:**
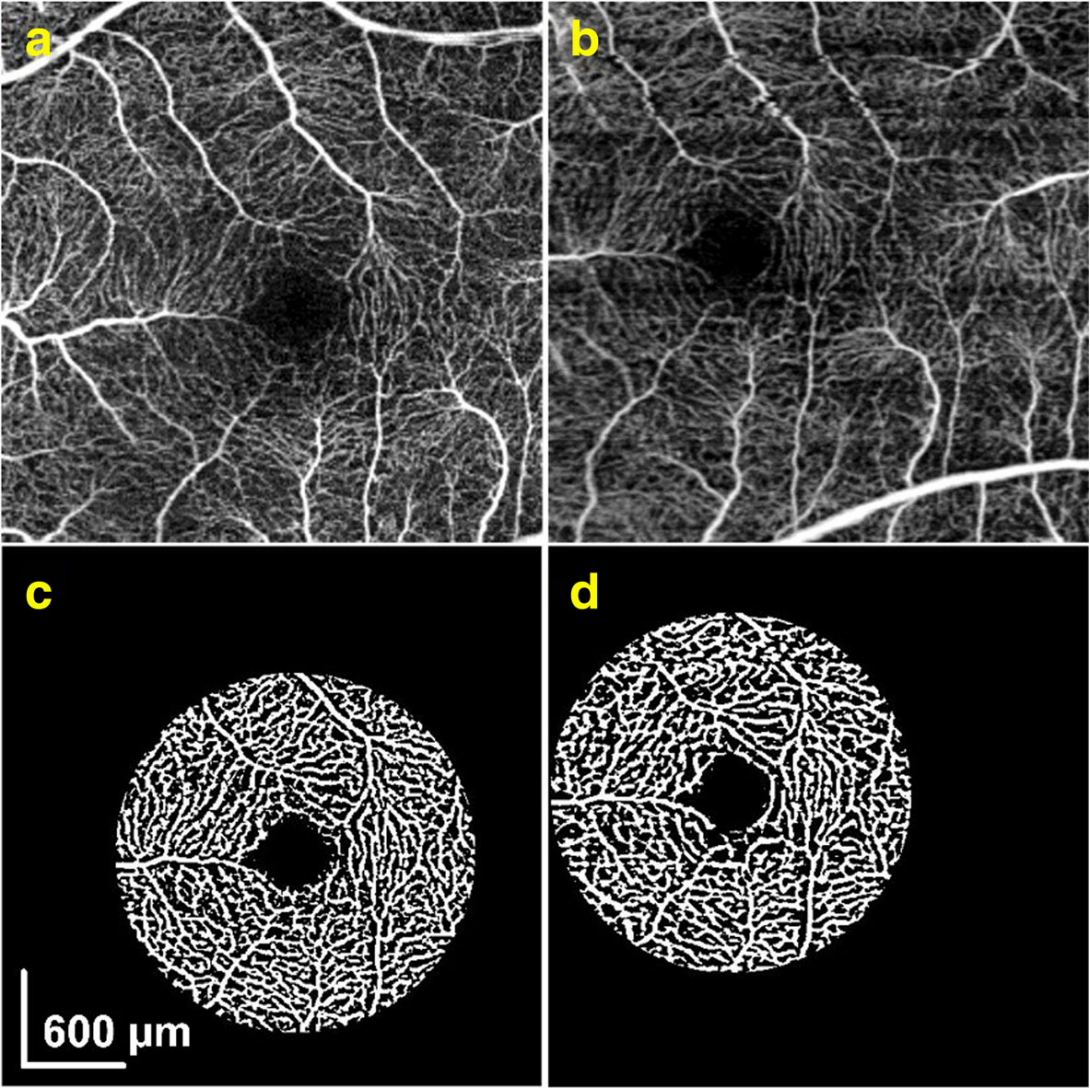
Representative OCT angiograms captured by (**a**) SD- and (**b**) SS-OCTA from the same subject. After co-registering (**a** and **b**), a maximum circular region within two images was identified that covers the same area of the fundus, upon which vessel binarization was applied, resulting in (**c** and **d**) for the SD- and SS-OCTA images, respectively. The vessel density was finally calculated from this common circular region for comparison. The avascular zone was excluded

In addition to structure-based signal strength comparison, angiographic capacities were compared between SD- and SS-OCTA in the retinal layer. For retinal layer, maximum intensity projection *en face* images were used for vessel density measurements; both the 3 × 3 mm and 6 × 6 mm scans from SD- and SS-OCTA were analyzed. Registration between SD- (Fig. 2a) and SS-OCTA (Fig. 2b) angiograms was carried out prior to the comparison, resulting in an identical circular region centered on the central fovea (Fig. 2c and Fig. 2d). From this circular region, vessel density was calculated through vessel binarization and excluding the foveal avascular zone [31].

### Signal strength difference and CSR volume measurements

To investigate how CSR affects the imaging capability of SD- and SS-OCTA, both the 3 × 3 mm and 6 × 6 mm scans from SD- and SS-OCTA were analyzed. Firstly, SD- and SS-OCT signal strength differences, ∆S, were defined in each layer using Eq. (3):

3 $$ \Delta  S={S}_{ss}-{S}_{sd} $$

CSV measurements were acquired separately based on the 3 × 3 mm and 6 × 6 mm scans. CSR region was manually segmented on every OCT B-scan as mentioned in section “Normalization and segmentation”. CSV was then achieved by calculating the segmented 3D volume.

### Statistical analyses

Paired sample *t*-tests [32] were performed to detect differences in signal strength and vessel density between SD- and SS-OCTA for all groups (including 3 × 3 mm and 6 × 6 mm scans on SD- and SS-OCTA imaging, normal and CSR eyes). Linear regression [33] and Bland-Altman analyses [34–37] were performed to investigate the correlation between SD- and SS-OCTA on CSV measurement (including 3 × 3 mm and 6 × 6 mm scans). Linear regression was also performed to evaluate the relationship between *∆S* and CSV measurements. *P* values below 0.05 were considered statistically significant.

## Results

Seven eyes (OS: 4/7) from 5 CSR patients (Male: 2/5) and 7 normal eyes (OS: 4/7) from 7 volunteers (Male: 3/7) were imaged at the University of Washington Eye Institute from June 2016 to September 2017. Mean patient age was 60.5 years and ranged from 56 to 65 years.

### Structural signal strength comparisons

**Fig. 3:**
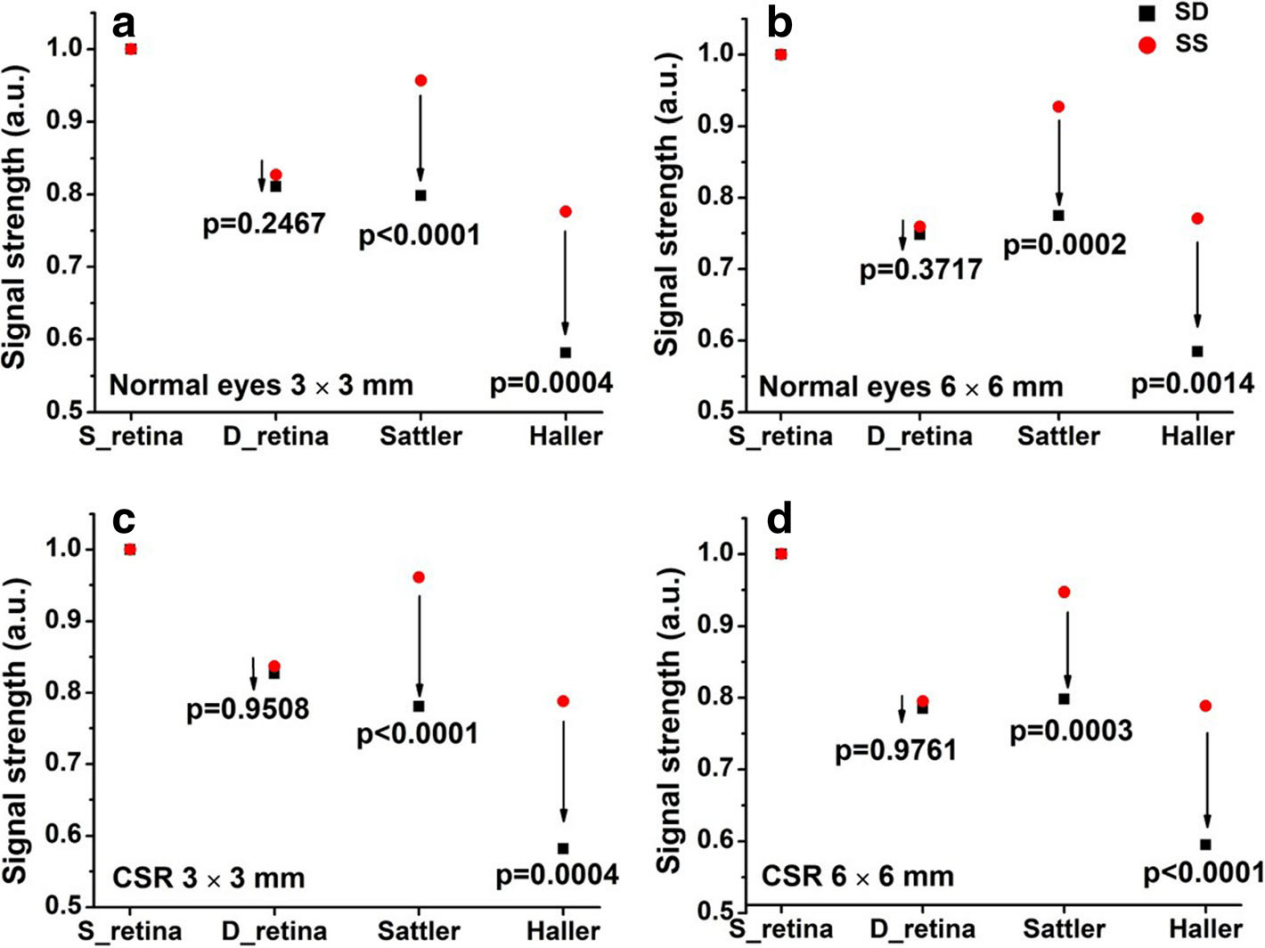
Signal strength comparisons between SD- and SS-OCT. Represented are the superficial retina, deep retina, Settler’s and Haller’s layers of (Top) normal and (Bottom) CSR eyes, imaged using scan patterns of (Left) 3 × 3 mm and (Right) 6 × 6 mm, respectively. S_retina denotes superficial retina, and D_retina denotes deep retina

Both SD- and SS-OCT systems display similar signal strength values in the deep retina layer of both normal and CSR eyes. However, a significant difference becomes apparent in the Sattler’s layer of both normal eyes (paired t-test, *p* < 0.0001 for 3 × 3 mm scans, and *p* = 0.0002 for 6 × 6 mm scans) and CSR eyes (paired t-test, *p* < 0.0001 for 3 × 3 mm scans, and *p* = 0.0003 for 6 × 6 mm scans). This trend continues in the Haller’s layer of normal eyes (paired t-test, *p* = 0.0004 for 3 × 3 mm scans, and *p* = 0.0014 for 6 × 6 mm scans) and CSR eyes (paired t-test, p = 0.0004 for 3 × 3 mm scans, and *p* < 0.0001 for 6 × 6 mm scans), as shown in Fig. 3.

### Angiographic capacity comparisons

Table 1 summarizes the measurements of retinal layer vessel density obtained by SD- and SS-OCTA, respectively. No significant differences were detected between either system for normal eyes (paired t-test, *p* = 0.33 for 3 × 3 mm scans, and *p* = 0.14 for 6 × 6 mm scans) and CSR eyes (paired t-test, *p* = 0.08 for 3 × 3 mm scans, and *p* = 0.15 for 6 × 6 mm scans).

**Table 1:** Comparison between SD and SS-OCTA of retinal layer vessel density

Datasets	Normal eyes				CSR eyes			
	3 × 3 mm, *n* = 7		6 x 6 mm, *n* = 7		3 × 3 mm, *n* = 7		6 x 6 mm, *n* = 7	
Instrument	SD-OCTA	SS-OCTA	SD-OCTA	SS-OCTA	SD-OCTA	SS-OCTA	SD-OCTA	SS-OCTA
Mean, %	47.44	47.92	45.15	46.32	41.83	42.71	42.49	42.86
SD	1.10	1.20	1.09	1.30	4.62	5.28	2.02	2.27
P value	0.33		0.14		0.08		0.15	

### Relationship between ***∆S*** and CSV

**Fig. 4:**
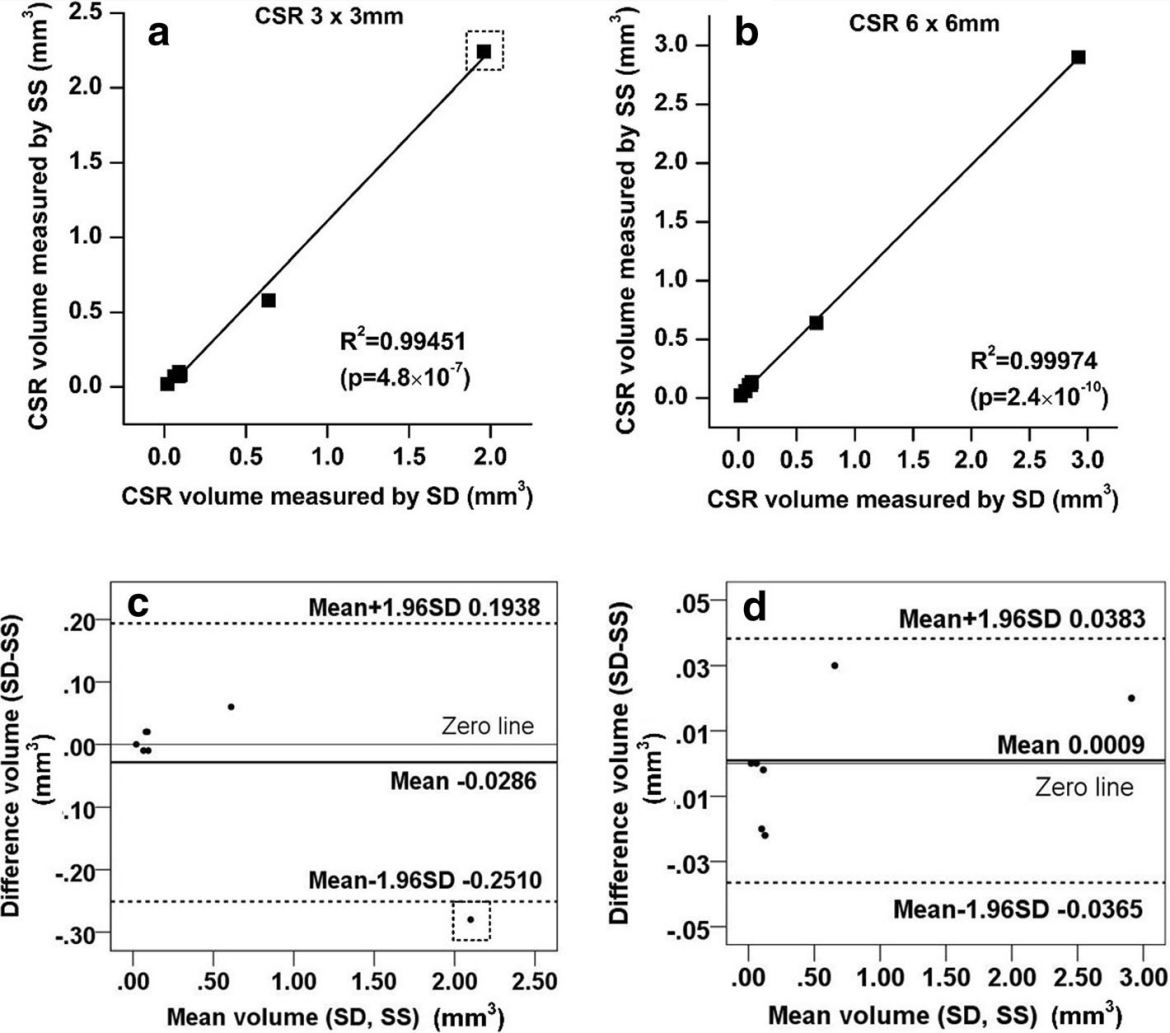
Central serous volume measurements using SD- and SS-OCTA. Both 3 × 3 mm (Left) and 6 × 6 mm (Right) fields of view are represented. **a** Comparison between SD- and SS-OCT measurements of CSV using 3 × 3 mm scan pattern using linear regression analysis, (**b**) Corresponding analysis from 6 × 6 mm scan pattern, (**c**) Bland-Altman analysis of (**a** and **d**). Bland-Altman analysis of (**b**). For the Bland-Altman analysis, the top and bottom dashed lines indicate the 95% limits of agreement (mean difference ± 1.96 standard deviation of the difference)

The CSV measurements show a significant correlation between SD- and SS-OCTA (linear regression analysis, *p* < 0.001 and R^2^ = 0.995 for 3 × 3 mm scans; *p* < 0.001 and R^2^ = 0.999 for 6 × 6 mm scans, Fig. 4a and b). Bland-Altman analysis shows there is a data point (highlighted with dashed square) exceeding the 95% limits of agreement (bottom dashed line, Fig. 4c). In this case, CSR volume is so substantial that a field of view (FOV) of 3 × 3 mm was insufficient to cover the entire CSR area while for 6 x 6 mm scans, the FOV is large enough to cover the entire CSR volume. The mean difference between SS- and SD-OCT measurements was just 0.0009 mm^3^ (Fig. 4d).

**Fig. 5:**
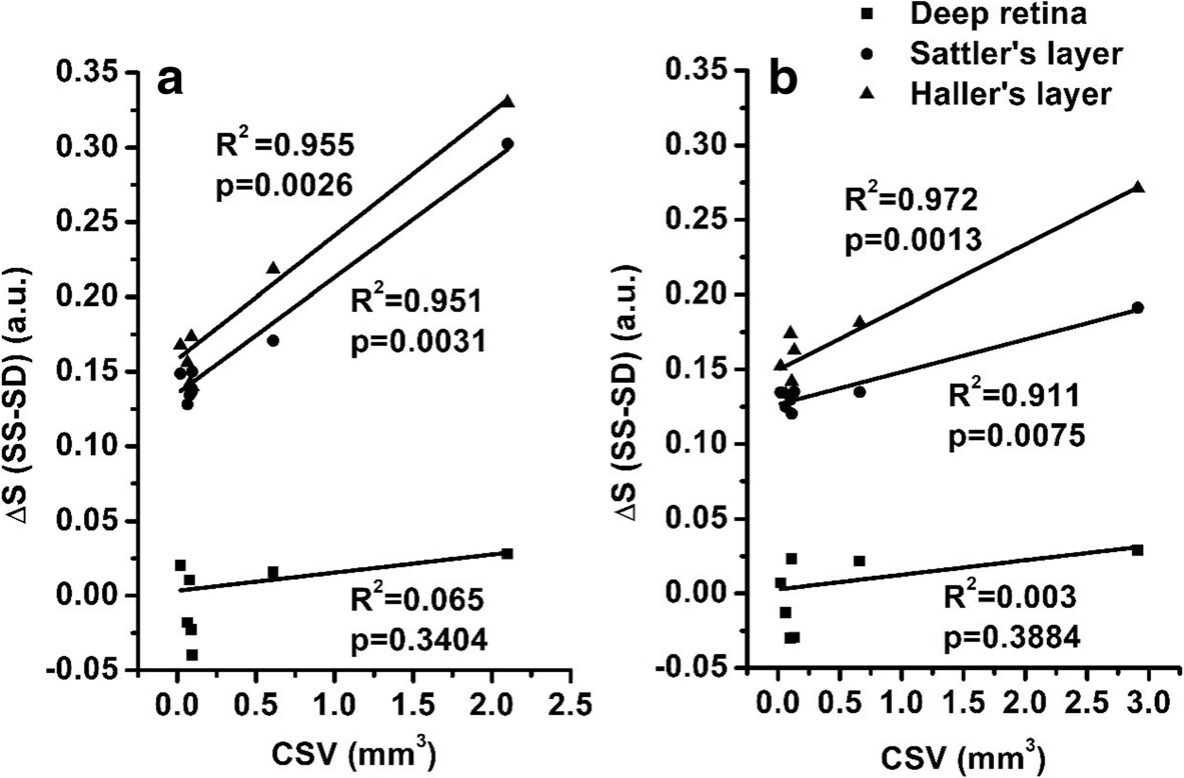
Analyses of signal strength difference, *∆S*, versus CSV measurements using SD- and SS-OCTA, (**a**) 3 × 3 mm, (**b**) 6 × 6 mm. Horizontal axis represents the measured CSV of each case, vertical axis represents the calculated *∆S* in deep retinal layer, Sattler’s layer and Haller’s layer, respectively

Linear regression analysis was carried out between *∆S* and CSV measurements (Fig. 5). In the deep retina layer, *p* = 0.340 and R^2^ = 0.065 for 3 × 3 mm scans, and *p* = 0.388 and R^2^ = 0.003 for 6 x 6 mm scans, indicating there is no correlation. *∆S* of the Sattler’s and Haller’s layers, however, both show strong correlations with CSV measurements. In the Sattler’s layer, *p* = 0.0031 and R^2^ = 0.951 for 3 × 3 mm scans, and *p* = 0.0075 and R^2^ = 0.911 for 6 x 6 mm scans. In the Haller’s layer, *p* = 0.0026 and R^2^ = 0.955 for 3 × 3 mm scans, and *p* = 0.0013 and R^2^ = 0.972 for 6 x 6 mm scans. In the deep layers beneath the CSR area, *∆S* linearly increases as CSV increases.

## Discussion

As a noninvasive technique, OCT is capable of generating in vivo cross-sectional volumetric images of anatomical structures with microscopic resolution (< 15 μm) in real-time, and it has been verified useful for our understanding of disease pathogenesis as well as clinical diagnosis and management of various ocular diseases [38–41]. Now, SD-OCT is already commercially available and has been widely used for quantitative examination of pathological eyes in patients [42] however, the short wavelength of light used by such a device reduces its light penetration into deep tissue, subsequently reducing its ability to accurately evaluate deep tissue. To overcome such a limitation, enhanced depth imaging (EDI)-OCT [43] was introduced to visualize the structural information within cross-sectional images representing regions below the RPE layer. Yet, current EDI-OCT only provides cross-sectional scans and averaging is often carried out on many repeated single-location B-frames by setting the choroid adjacent to the zero-delay line where the system has maximal sensitivity. These limitations make 3D scanning difficult. More recently, SS-OCT devices are becoming popular as they have shown to improve the visualization of structure and flow due to an improved sensitivity roll-off and attenuation of OCT signal from deeper tissues. A number of prior studies were designed to compare SD- with SS-OCT for use in assessing ophthalmologic parameters [20–22, 44]. One study demonstrated that the choroidoscleral interface can be clearly visualized in 19/19 (100%) of eyes imaged by SS-OCT, compared to 14/19 (73.6%) and 13/19 (68.4%) eyes imaged by SD-OCT, with and without EDI, respectively [21]. In another study by Matsuo et al., SS-OCT was shown to measure a thicker sub-foveal choroid than that measured by SD-OCT instruments on normal eyes (273.2 μm by Spectralis-SD-OCT, 269.1 μm by Topcon-SD-OCT and 280.5 μm by DRI-SS-OCT) [44]. As a further development to OCT technology, OCTA was introduced to the field of clinical ophthalmology, which can provide blood flow information via motion contrast algorithm. It utilizes the flowing red blood cells as an intrinsic contrast agent to generate blood flow signals thereby allowing the visualization of functional vascular networks without the need for a dye injection. Several studies were also conducted to compare SD- and SS-OCTA on vessel imaging e.g. the CNV quantification [18, 19]. The results revealed that SS-OCTA measured significantly larger CNV sizes than SD-OCTA. Considering these studies, SS-OCTA did appear to encourage improved light penetration into deeper tissues, which in turn resulted in improved OCT/OCTA signal detection. This was thought to be attributed to several factors. Firstly, SS-OCTA operates at a longer wavelength, ~ 1050 nm, compared with SD-OCTA, ~ 840 nm, which allows more light to penetrate deeper tissue due to the reduced scattering properties of tissue. Secondly, a balanced detector was used within the SS-OCTA system instead of the charge-coupled device (CCD) camera used in the SD-OCTA system, giving a higher signal to noise ratio (SNR). Furthermore, the narrow linewidth of swept-laser source corresponds to a relatively longer coherence length, leaving SS-OCTA with a delayed sensitivity roll-off thus boosting the detection of the signals from deep regions when compared with SD-OCTA. Taken together, SS-OCTA is advantageous over SD-OCTA when acquiring structural and flow information from deeper tissues.

CSR is characterized by an acute or chronic form of retinal disorder, which involves a detachment of the neurosensory retina secondary to one or more focal fluorescein angiographic leaks at the level of the RPE layer. Although acute CSR can sometimes recur, it generally resolves spontaneously with minimal sequelae. However, chronic CSR can result in widespread RPE damage. These patients have longstanding subretinal fluid that cannot be absorbed efficiently because of choroidal dysfunction and loss of RPE. The presence of fluid leads to photoreceptor death and may result in a series of complications in deep region e.g. the CNV. Considering that central serous bump further extends the depth of the lesion, CSR can be a potential disease group that could benefit from improved deep tissue imaging. Thus, comparisons between SD- and SS-OCTA using structural and angiographic features associated with CSR were addressed in this study. The results of signal strength comparisons suggest that there is no significant difference between either OCT method in terms of information quality when imaging superficial tissues, however, SS-OCTA can detect more structural signals from deep tissue compared to SD-OCTA either in normal subjects or CSR patients. In CSR patients, we further evaluated how the CSV affects SD- and SS-OCTA’s imaging abilities. Our data has revealed that said advantages, *∆S*, of using SS-OCTA over SD-OCTA linearly increases with the increase of CSV, meaning the limiting influences of the central serous bump are more detrimental when using SD-OCTA. This is an important conclusion. Thus, SS-OCTA may be recommended for accurate diagnosis of CSR patients, especially in big CSV cases.

There are still a few limitations in our study. Although our sample size is small, we find it to be acceptable because signal strength was shown to be a robust parameter when comparing SD- and SS-OCTA. The results gave a clear conclusion that SS-OCTA detected a stronger structural signal strength in deep choroid compared to SD-OCTA, suggesting that SS-OCTA has an advantage over SD-OCTA on angiographic imaging in deep layers. Yet, a larger sample size is necessary if the study is required to compare signals beyond the RPE layer e.g. in the choriocapillaris [45–48]. In addition, the sample resolutions of 3 × 3 mm and 6 × 6 mm scanning protocols are different and unchangeable between SD- and SS-OCTA due to the highly integrated systems used in our study, which might have an impact on the final image quality, but it is unclear how differences in sample resolution might have an effect on the results.

## Conclusion

In the present study, signal strength has been shown to be a robust parameter when comparing SD- and SS-OCTA, based on evidence that SS-OCTA is able to improve deep tissue imaging compared to SD-OCTA, especially when CSR is present. The possible clinical advantage of SS-OCTA in terms of diagnosis and disease monitoring should be reviewed in further studies with a large sample size and comprising various retinal and choroidal diseases.
